# Human-specific dual regulations of FXR-activation for reduction of fatty liver using *in vitro* cell culture model

**DOI:** 10.3164/jcbn.18-80

**Published:** 2018-11-28

**Authors:** Teruo Miyazaki, Akira Honda, Tadashi Ikegami, Takashi Iida, Yasushi Matsuzaki

**Affiliations:** 1 Joint Research Center, Tokyo Medical University Ibaraki Medical Center, 3-20-1 Chuo, Ami, Inashiki, Ibaraki 300-0395, Japan; 2 Department of Internal Medicine, Division of Gastroenterology and Hepatology, Tokyo Medical University Ibaraki Medical Center, 3-20-1 Chuo, Ami, Inashiki, Ibaraki 300-0395, Japan; 3 Department of Chemistry, College of Humanities and Sciences, Nihon University, 3-25-40 Sakurajosui, Setagaya-ku, Tokyo 156-8550, Japan

**Keywords:** fatty liver, nuclear receptor ligand, human-specific crosstalk, bile acids, lipolysis

## Abstract

Nuclear receptor farnesoid X receptor activation inhibits fatty acid synthesis through the liver X receptor-α-sterol regulatory element binding protein-1c pathway universally in animals, but also has human-specific crosstalk with the peroxisome proliferator-activated receptor-α. The effects of farnesoid X receptor-ligands on both the synthesis and degradation of fatty liver through nuclear receptor-related regulation were investigated in both human and murine hepatocytes. A fatty liver culture cell model was established using a synthetic liver X receptor-α-ligand (To901317) for both human and mouse non-neoplastic hepatocytes. The hepatocytes were exposed to natural or synthetic farnesoid X receptor-ligands (bile acids, GW4064, obeticholic acid) together with or after To901317. Cellular triglyceride accumulation was significantly inhibited by the farnesoid X receptor-ligands along with inhibition of lipogenic genes and up-regulation of farnesoid X receptor-target small heterodimer partner in both human and mouse cells. The accumulated triglyceride was significantly degraded by the farnesoid X receptor-ligands only in the human cells accompanied with the up-regulations of peroxisome proliferator-activated receptor-α and fatty acid β-oxidation. Farnesoid X receptor-ligands can be therapeutic agents for treating human fatty liver through dual effects on inhibition of lipogenesis and on enhancement of lipolysis.

## Introduction

Fatty liver, also known as hepatic steatosis, is characterized by the deposition of triglyceride (TG) as lipid droplets in the cytoplasm of hepatocytes (>55 mg/g of liver or >5% TG droplets in the liver).^(1)^ In addition to the various etiological causes, fatty liver is associated with diabetes mellitus and metabolic syndrome, and is the early stage of nonalcoholic fatty liver disease (NAFLD) that could progress to non-alcoholic steatohepatitis (NASH), liver cirrhosis, and cancer. The prevalence of diseases associated with fatty liver is increasing every year in the world, particularly in the developed countries. However, the early stage as fatty liver has not been received much attention for treatment because the state is not usually problematic due to lack of symptoms or pain. Furthermore, effective or useful therapies have been untested for this early manifestation.

**Fig. 1:**
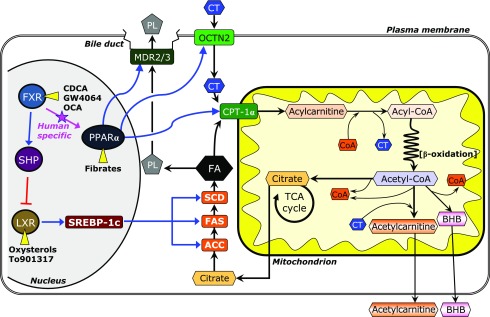
Regulations of fatty acid metabolism, including synthesis, degradation, and secretion through nuclear receptors in hepatocytes. LXRα activated by natural ligand oxysterols and synthetic ligands induces the transcriptional factor SREBP-1c, and consequently, fatty acid biosynthesis from citrate is increased due to up-regulated gene expressions of fatty acid synthesis enzymes (ACC-1, FAS, and SCD-1). FXR activation promotes multilaterally the decrease of fatty acid levels through the inhibition of biosynthesis, and the enhancements of degradation and excretion of fatty acids. FXR activated by natural ligand bile acids and synthetic ligands inhibits SREBP-1c via up-regulation of SHP. The activated FXR also enhances nuclear receptor PPARα that can be activated by natural ligand fatty acids and synthetic ligand. The activated PPARα increases its target gene expressions (OCTN2 and CPT-1α). This crosstalk of FXR and PPARα limits to humans. In addition, activated PPARα also enhances the excretion of fatty acids as phospholipids (PL) into the bile via up-regulation of the gene expression of MDR protein.

In the liver, the genes involved in lipogenesis are essentially regulated by the sterol regulatory element factor binding protein-1c (SREBP-1c) that is produced by the activation of liver X receptor-α (LXRα: NR1H3), a nuclear receptor for cholesterol derivatives, oxysterols (Fig. 1).^(2–4)^ The LXRα-SREBP-1c pathway is directly inhibited by the atypical nuclear receptor small heterodimer partner (SHP; NR0B2) that is a target of the farnesoid X receptor (FXR; NR1H4).^(5,6)^

FXR, a member of the nuclear hormone receptor superfamily and a bile acid-responsive transcription factor, plays key roles not only in the regulation of bile acid synthesis and enterohepatic recirculation but also in both lipid and glucose metabolism.^(7)^ FXR-ligands have recently been the focus as the molecular target of pharmacological therapies for hepatobiliary^(8–10)^ and gastrointestinal diseases.^(11)^ Obeticholic acid (6α-ethyl derivative of chenodeoxycholic acid, OCA^(12,13)^), a first-in-class selective FXR-ligand, has been evaluated in clinical trials of the patients with type II diabetes mellitus, NASH, and primary biliary cholangitis (PBC).^(14–18)^ These clinical studies demonstrated the excellent effectiveness of OCA treatment on cholestasis, insulin sensitivity, inflammation, and fibrosis in these patients. In addition, oral OCA administration improved hepatic steatosis in patients with non-cirrhotic, non-alcoholic steatohepatitis in one clinical trial,^(19)^ and this effect was likely due to inhibition of lipogenesis through the FXR-SHP-SREBP-1c cascade (Fig. 1).^(5,6)^ However, there are no studies on the expression of SREBP-1c-regulated lipogenic genes in the liver tissues of the patients treated with the FXR-ligand.

In the studies on various rodent models including Zucker (*fa*/*fa*) rats,^(20)^ ApoE^−/−^ mice,^(21)^ LDL receptor ^−/−^ mice,^(22)^ and obese *ob*/*ob* mice,^(23)^ the elevated mRNA expression of the essential gene transcriptional factors in lipogenesis, including SREBP-1c and its target genes [acetyl-CoA carboxylase-1 (ACC-1), fatty acid synthase (FAS), malic enzyme, stearoyl-CoA desaturase-1 (SCD-1), and etc.] as well as a gluconeogenetic enzyme (phosphoenolpyruvate carboxykinases) in the liver tissues were decreased by OCA and other synthetic FXR-ligands, GW4064 and WAY-362450, accompanied with decreased hepatic TG levels. Furthermore, steatosis was reduced by OCA or GW4064.^(24)^ Therefore, the inhibitory effects of FXR-ligands on the hepatic steatosis in animal models are associated with decreased lipogenesis through the FXR-SHP-SREBP-1c cascade.

Likewise, the FXR-SHP-SREBP1c cascade is the mechanism of the attenuation of steatosis in the patients of the FXR-ligand treatment. However, there is a possibility that the improvement in steatosis by FXR-ligands are due to not only to inhibition of lipogenesis but also to enhancement of lipolysis, because there is molecular crosstalk between the FXR and peroxisome proliferator-activated receptor-α (PPARα; NR1C1) (Fig. 1). PPARα is highly expressed in the liver and is a principal lipid catabolism regulator in fatty acid transport and oxidation. Recent study showed that the PPARα activation could improve hepatic steatosis by promoting of fat oxidation together with the inhibiting of lipogenesis through SREBP1c activation in the soy isoflavone-treated high fat diet NAFLD model rats.^(25)^ It is up-regulated by natural ligands, fatty acids, and synthetic ligands, such as fibrates.^(26,27)^ Therefore, FXR-ligand treatment is suggested to activate the PPARα-regulated lipolysis in fatty liver. Noteworthy, the crosstalk between the FXR and PPARα is human-specific because of the presence of an FXR response element in the PPARα promoter (α-FXR response element) that is present in humans, but not in rodents.^(28)^ Therefore, the effect of an FXR-ligand on PPARα-regulated gene expressions in the liver cannot be evaluated in the animal models, and has not been studied in the steatotic livers of the patients in the clinical trials.

Therefore, culture cell model of fatty liver as an experimental tool is useful to better understand the gene responses in both lipogenesis and lipolysis of the liver under FXR-ligand treatment. As fatty liver models of culture cell, the treatment of fatty acids has been well established in the primary hepatocytes and cell lines.^(29,30)^ However, the fatty acids-induced fatty liver models are unsuitable for the evaluation of these points, because fatty acids are natural PPARα ligand. Furthermore, there are some restrictions and inconveniences in the primary hepatocytes on the limited and erratic supply, the significant inter-individual variability in the gene expression, and the culture duration and maintenance. The present study firstly attempted to establish a novel hepatic steatosis culture cell model for both human and mouse using non-malignant hepatic cell lines treated with natural and synthetic LXRα ligand (oxysterols,^(31)^ To901317^(32)^). Then, the effects of FXR-activation on lipogenesis and on lipolysis were compared between human and mouse in the newly established fatty liver culture cell model using natural and synthetic ligands (bile acids, GW4064, OCA).

## Materials and Methods

### Chemicals

Ursodeoxycholic acid (UDCA), chenodeoxycholic acid (CDCA), 4β-hydroxycholesterol (4β-HC), 22R-hydroxycholesterol (22R-HC), 25-hydroxycholesterol (25-HC), and 24S,25-epoxycholesterol (24S,25-EC) were purchased from Steraloids (Wilton, NH). To901317 {*N*-(2,2,2-trifluoro-ethyl)-*N*-[4-(2,2,2-tri-fluoro-1-hydroxy-1-trifluoromethyl-ethyl)-phenyl]-benzenesulfonamide}, acetyl-l-carnitine HCl, and sodium d-β-hydroxybutyrate (BHB) were purchased from Sigma-Aldrich Chemical Co. (St. Louis, MO), and GW4064 (3-[2-[2-Chloro4-[[3-3(2,6-dichlorophenyl)-5-(1-methylethyl)-4-isoxazolyl]methoxy] phenyl]ethenyl]benzoic acid) were purchased from Tocris Cookson Inc. (Ellisville, MO). Acetyl-l-[^2^H_3_]carnitine HCl was purchased from C/D/N Isotopes Inc. (Quebec, Canada). Sodium dl-BHB-^13^C_4_ was obtained from Taiyo Nippon Sanso Co. (Tokyo, Japan), and 2-pyridinemethanol and 2-methyl-6-nitrobenzoic anhydride were obtained from Tokyo Kasei Kogyo (Tokyo, Japan). l-carnitine and 4-dimethylaminopyridine were obtained from Wako Pure Chemical Industries (Osaka, Japan). Fenofibric acid (FA) was supplied by ASKA Pharmaceutical. Co., Ltd. (Tokyo, Japan). OCA was a kind gift from Dr. Pelliciari R. (University of Bologna, Bologna, Italy), and from Dr. Hofmann A. (University of California, Berkeley, CA). These chemical reagents except for the bile acids were dissolved in ethanol and added to medium at 1:100 dilution. Additional reagents and solvents were of analytical grade.

### Cultured hepatocytes

Non-tumor cultured hepatocyte cell lines and primary cultured hepatocytes of mouse and human were used for experimental steatosis culture model. AML12 cells, a differentiated, non-transformed cell line that was derived from transforming growth factor α over-expressing transgenic mice (ATCC, Manassas, VA),^(33)^ were cultured in a 1:1 mixture of Dulbecco’s modified Eagle’s medium (DMEM) and Ham’s F12 medium (DMEM/F12, Invitrogen Japan KK, Tokyo, Japan) containing 3.151 g/L glucose, supplemented with Insulin-Transferrin-Selenium-X (ITS, final concentration; 5 µg/ml in each, GIBCO®, Invitrogen), 40 ng/ml dexamethasone (BioVision, Inc., Mountain View, CA), and 10% fetal bovine serum (FBS). Fa2N-4 cells, a human cell line immortalized by transfection with SV40 large T antigen (MultiCell Technologies Inc., Woonsocket, RI),^(34)^ were cultured in DMEM containing 4.5 g/L glucose, with 10% FBS. Primary hepatocytes of mouse and human (Life Technologies^TM^, Thermo Fischer Scientific Inc., Rockford, IL) were seeded on a collagen coated 24-well plate as 3.0 × 10^5^ cells/well in the plating medium [Williams’ Medium (Invitrogen Japan KK) with the Hepatocyte Plating Supplement Pack (CM3000, Invitrogen Japan KK) containing 5% FBS, 0.1 µM dexamethasone, 100 U/ml Penicillin/Streptmycin, 4 µg/ml human recombinant insulin, 2 mM GlutamaxTM, 15 mM HEPES]. After 6 h, the medium was changed to the maintenance medium [Williams’ Medium with the Cell Maintenance Supplement Pack (CM4000, Invitrogen Japan KK) containing 0.1 µM dexamethasone, 50 U/ml Penicillin/Streptmycin, 6.25 µg/ml ITS, 1.25 mg/ml bovine serum albumin (BSA), 5.35 µg/ml linoleic acid, 2 mM GlutamaxTM, 15 mM HEPES]. After overnight culture, the primary hepatocytes were used for following experiments. All cells were incubated at 37°C in a humidified incubator containing 5% CO_2_ and 95% air.

### Generation of a steatosis model using non-tumor hepatocyte cell lines

Sub-confluent AML12 cells were exposed to either 10 µM oxysterols (4β-HC, 22R-HC, 25-HC or 24S,25-EC) or 1 µM To901317, for 4 days. Each reagent was dissolved in ethanol and was added to fresh medium at 100-fold dilution (1%). Although 1% ethanol had no detectable influence on cell growth, the same concentration of ethanol was also added to control wells. In addition, the effectiveness of To901317 on lipogenesis was evaluated in dose- and time-dependent manners. The Fa2N-4 cells were also exposed to 1 µM To901317 for 4 days. After the exposure period, the cells were collected and used for analyses of lipogenesis-related gene expression and intracellular TG accumulation.

### Experimental protocols

AML12 and Fa2N4 cells were seeded in 6-well plates and cultured until sub-confluent to investigate the effect of FXR-ligands on the lipogenesis. Then, the cells were exposed to each FXR-ligand together with 1 µM To901317 for 4 days. The used FXR-ligands used for the study were 50 µM UDCA, 50 µM CDCA, 1 µM GW4064, and 1 µM OCA. After exposure for 4 days, gene expression for lipogenesis and intracellular TG levels were analyzed by the methods discussed below. For the evaluation of effectiveness of FXR-ligands on TG catabolism, the cell lines were first exposed to 1 µM To901317 for 4 days. Then, the cells were washed twice with PBS and cultured with each FXR-ligand or a synthetic PPARα-ligand (1 µM FA) for 3 days. Then, gene expression for TG catabolism and intracellular TG levels were assayed.

The primary hepatocytes of mouse and human were exposed to 1 µM To901317 with 1 µM GW4064 or 1 µM OCA for 24 h.

### RNA measurement

Total RNA was extracted from the cells using the RNeasy Plus Mini Kit (QIAGEN K.K., Tokyo, Japan). Reverse transcription was performed on 0.5~1.0 µg of total RNA using the PrimeScript^®^ RT reagent Kit (TAKARA Bio. Inc. Shiga, Japan). Real-time quantitative PCR was performed on cDNA aliquots with the FastStart DNA Master SYBR Green I and the LightCycler (Roche Diagnostics, Mannheim, Germany). The sequences of the oligonucleotide primer pairs used to amplify the mRNA in the mouse and human cells are shown in Table 1. PCR amplification began with a 10 min pre-incubation step at 95°C, followed by 40 cycles of denaturation at 95°C for 10 s, annealing at 62°C for 10 s, and elongation at 72°C (Table 1). The relative concentration of PCR product derived from the target gene was calculated using the LightCycler System software. A standard curve for each run was constructed by plotting the crossover point against the log concentration. The concentration of target molecules in each sample was then calculated automatically by reference to this curve (*r* = −1.00), and the results were standardized to the expression of β-actin. The specificity of each PCR product was assessed by melting curve analysis.

### TG assays

Cellular TG levels were measured using the Triglyceride E-Test Wako (Wako Pure Chemical Industries). After each experiment, the cells were washed twice with PBS, and then, collected into a 0.5 ml tube with 100 µl PBS. The collected cells were sonicated, and used for the assay and for measurement of total protein using the Pierce^®^ BCA Protein Assay Kit (Thermo Fischer Scientific Inc.). Cellular TG concentration was expressed as per total protein level. The cellular TG was also stained with oil-red using the Lipid Assay Kit (Primary Cell Co., Ltd., Hokkaido, Japan). For histology, the cells were fixed with 4% paraformaldehyde overnight, and the cellular TG was stained with oil-red following two washes with PBS.

### β-Oxidation activity

AML12 and Fa2N-4 cells seeded in 6-well plates were cultured in the growth media mentioned above. When the cells were confluent, the media were replaced with a specific medium to derive the β-oxidation reaction: serum-free DMEM containing 1.0 g/L glucose, 1 mM l-carnitine, and 200 µM palmitic acid with 10% (*w/v*) fatty-free BSA. For the β-oxidation-derivative medium, either 50 µM UDCA, 50 µM CDCA, 1 µM GW4064, 1 µM OCA, or 1 µM FA was also added. After incubation for 4 h, the culture media were collected to measure the concentrations of acetylcarnitine and BHB that are metabolites excreted from the cells due to β-oxidation.^(35)^

β-Hydroxybutyrate concentration in the culture medium was assayed as described previously.^(36)^ Briefly, 5 µl of the medium was mixed with 100 ng of sodium dl-BHB-^13^C_4_ in 100 µl of acetonitrile-water (19:1, *v/v*), and deproteinized liquid phase was collected and evaporated to dryness. After derivatization of BHB into 2-pyridinemethanol esters, an aliquot was assayed by the HPLC-electrospray ionization (ESI)-MS/MS system.

Acetylcarnitine in the medium was quantified by the HPLC-ESI-MS/MS system as described previously^(37)^ with some modifications of a method reported by Ghoshal *et al.*^(38)^ Ten µl of the medium was mixed with 5 ng of acetyl-l-[^2^H_3_]carnitine HCl in 100 µl of acetonitrile-water (19:1, *v/v*), and the deproteinized liquid phase was collected and evaporated to dryness. The residue was redissolved in 0.1% formic acid solution, and an aliquot was analyzed by the HPLC-ESI-MS/MS system. The chromatographic separation was performed using a Hypersil GOLD a Q column (150 × 2.1 mm, 3 µm, Thermo Fisher Scientific) at 40°C. The mobile phase was methanol-water (1:9, *v/v*) containing 0.1% formic acid at a flow rate of 200 µl/min. The MS/MS conditions were as follows: spray voltage, 3,000 V; vaporizer temperature, 450°C; sheath gas (nitrogen) pressure, 50 psi; auxiliary gas (nitrogen) flow, 15 arbitrary units; ion transfer capillary temperature, 220°C; collision gas (argon) pressure, 1.0 mTorr; collision energy, 15 V; ion polarity, positive; and SRM, *m*/*z* 204 g *m*/*z* 85 for the acetylcarnitine and *m*/*z* 207 g *m*/*z* 85 for the [^2^H_3_] variant.

### Cell viability

The influence of chemical reagents used on the cell viability was evaluated by the MTT assay.^(39)^ AML12 and Fa2N-4 cells seeded on a 24-well plate were exposed to either 1 µM each of To901317, GW4064, or OCA, or to 10–100 µM each of UDCA and CDCA for 24 h.

### Statistical analysis

All presented data are the mean ± SD from at least three independent experiments. Significant differences were determined by the unpaired student’s *t* test or one-way ANOVA post hoc Bonferonni’s test for comparisons between two groups or among multiple groups, respectively. The statistical analyses were performed using Prism software (GraphPad Software, Inc., La Jolla, CA).

## Results

### Steatosis model of cultured hepatocytes treated with LXRα-ligand

**Fig. 2:**
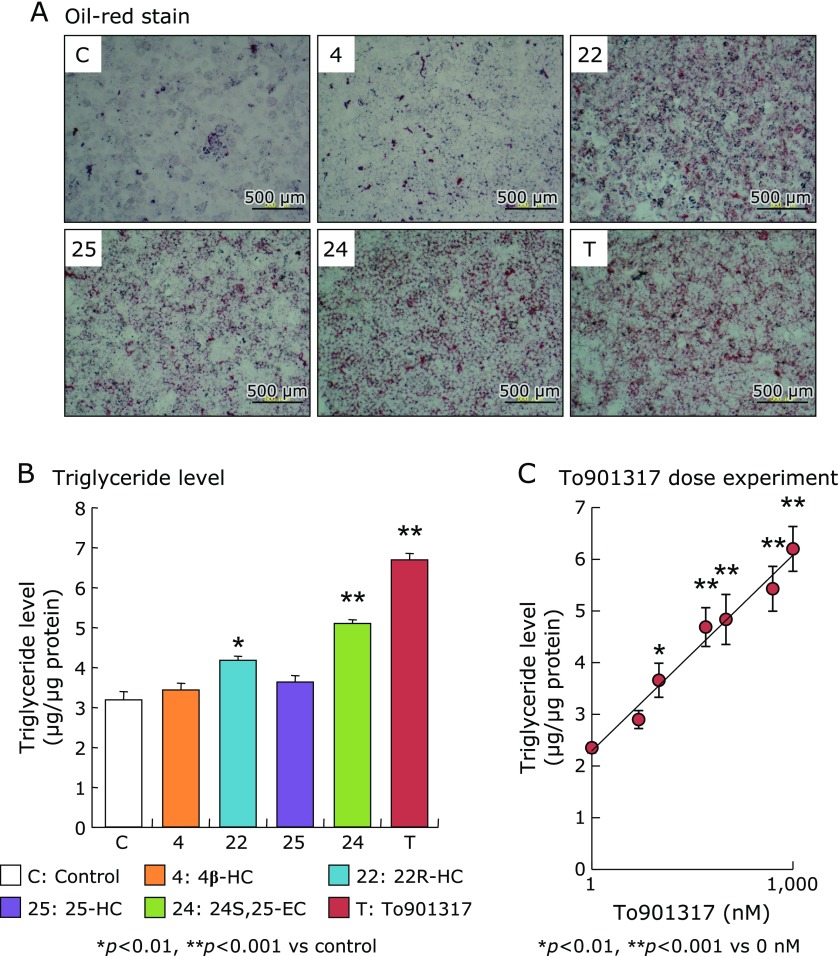
Oil-red stain image (A) and quantified intracellular triglyceride level (B) in AML12 cell after the exposure of either 10 µM of oxysterols (4β-HC, 22R-HC, 25-HC, or 24S,25-EC) or 1 µM of To901317 for 4 days. (C) shows triglyceride level in AML12 cell after exposure to various concentrations of To901317 (0, 10, 25, 50, 100, 500, and 1,000 nM) for 4 days. Triglyceride level in the cells was measured by the triglyceride test kit Wako and expressed as per total protein level, and was also stained with oil-red. Data in the (B) and (C) are shown as the mean ± SD. Statistical difference was carried out by one-way ANOVA Bonferroni’s post hoc test.

In AML12 cells exposed to various types of oxysterol as natural LXRα-ligand or a synthetic LXRα-ligand To901313 for 4 days, markedly increase of intracellular TG accumulation evaluated by the oil-red stain assay was observed in exposing to 22R-HC, 25-HC, 24S,25-EC, and To901317 (Fig. 2A). In the quantification assay, intracellular TG levels were significantly increased in the cells exposed to 22R-HC, 24S,25-EC, and To901317 compared with that of the control (Fig. 2B). Cellular TG level was increased in a dose-dependent manner up to 1 µM To901317 that had the most potent ligand activity among the examined LXRα-ligands (Fig. 2C).

**Fig. 3:**
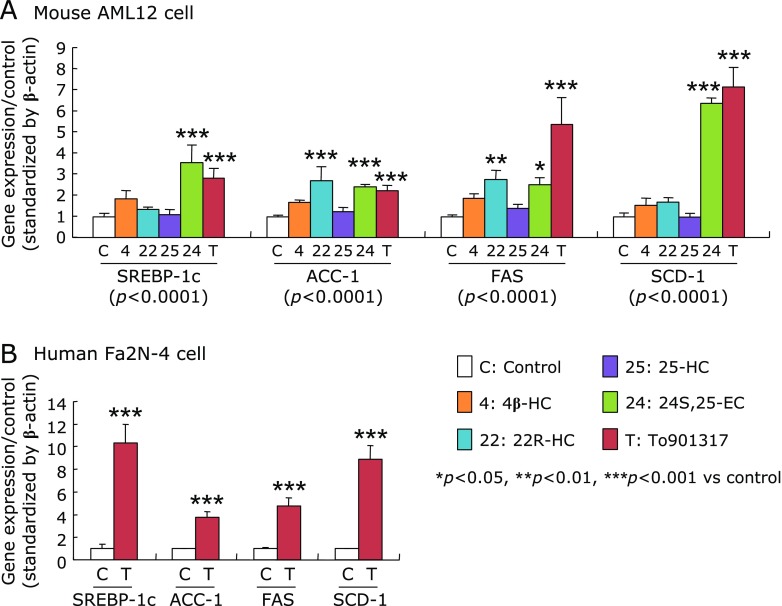
The mRNA levels of SREBP-1c and its target genes in AML12 cells (A) and human Fa2N-4 cells (B), exposed to either natural or synthetic LXRα-ligands. AML12 cells were treated with either 10 µM of each oxysterol or 1 µM To901317, and Fa2N-4 cells were treated with or without 1 µM To901317 for 4 days. The mRNA levels were measured by quantitative real-time PCR and expressed as the relative to control after standardization by β-actin. Data are shown as the mean ± SD. Statistical differences were carried out by one-way ANOVA Bonferroni’s post hoc test, or unpaired student’s *t* test. Values in the parenthesis of (A) show *p* values of one-way ANOVA test.

In AML12 cells exposed to oxysterols or To901317 for 4 days, the mRNA levels of SREBP-1c and all examined SREBP-1c target genes (ACC-1, FAS and SCD-1) were significantly increased by 24S,25-EC and To901317 treatments compared to those in the controls (Fig. 3A). In addition, 22R-HC treatment significantly up-regulated mRNA levels of ACC-1 and FAS. Since the LXRα-activations were greater in To901317 than in the oxysterols in AML12 cells, To901317 was selected for further study of Fa2N-4 cells. Similar to AML12 cells, the mRNA levels of SREBP-1c and its target genes in the Fa2N-4 cells were significantly elevated by To901317 for 4 days (Fig. 3B).

### Anti-lipogenic and pro-lipolytic effects of FXR-ligands

**Fig. 4:**
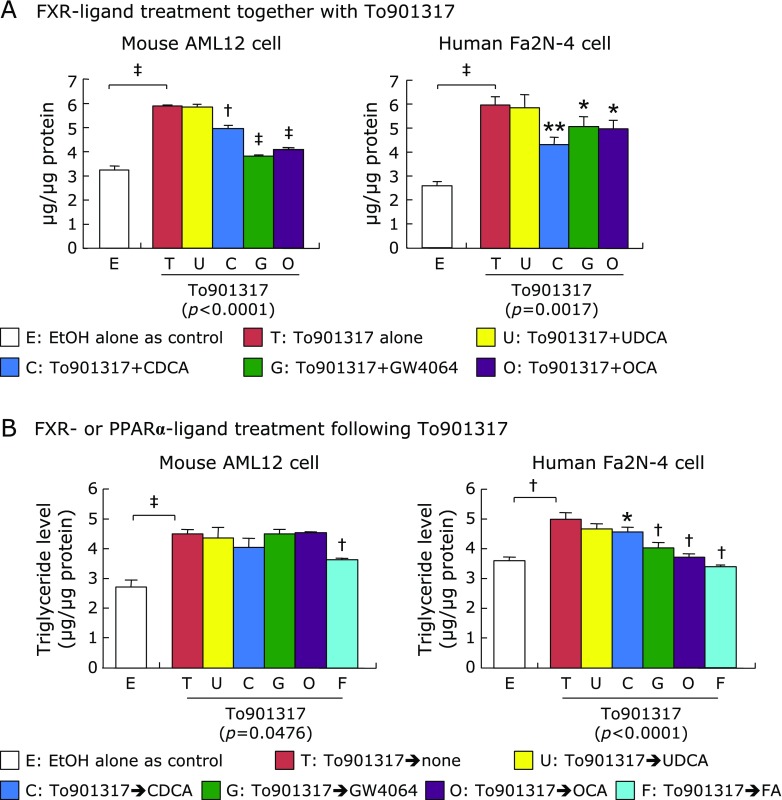
The effects of FXR-ligands on synthesis and catabolism of triglyceride in the cells exposed to To901317. (A) AML12 and Fa2N-4 cells were exposed to 1 µM of To901316 with or without 50 µM of natural ligand bile acids; UDCA and CDCA, or 1 µM of synthetic FXR-ligands; GW4064 and OCA, for 4 days. (B) AML and Fa2N4 cells were exposed to the natural FXR-ligand bile acids, synthetic FXR-ligands, or synthetic PPARα-ligand; FA, for 3 days following To901316 treatment for 4 days. Triglyceride level was expressed as the concentration per total protein level. Data are shown as the mean ± SD. Statistical differences were analyzed by unpaired Student’s *t* test between the control and To901317 alone or one-way ANOVA Bonferroni’s post hoc test between the To901317 alone and other ligand-treated conditions. *p<0.05, **p<0.01, ^†^*p*<0.001, ^‡^*p*<0.0001. Values in the parenthesis under graph show *p* values of one-way ANOVA test.

Using the established steatosis cultured cell model with To901317, the effect of FXR-ligand treatment on the TG levels was compared between the mouse AML12 and human Fa2N-4 cells. After cotreatments of either natural FXR-ligands (UDCA and CDCA) or synthetic FXR-ligands (GW4064 and OCA) together with To901317 for 4 days, cellular TG level was significantly reduced in both AML12 and Fa2N-4 cells by cotreatment of CDCA, GW4064, and OCA, but not UDCA, compared to that in the To901317 alone (Fig. 4A).

To evaluate the lipolytic effects of FXR activation on accumulated TG level, both cell lines were treated with the natural and synthetic FXR-ligands as well as synthetic PPARα-ligand (FA) in the absence of To901317 for 3 days in the steatosis cell model with To901317 for 4 days. In AML12 cells, the accumulated TG level was unchanged by any FXR-ligands, but significantly reduced by FA (Fig. 4B). In contrast, cellular TG level in Fa2N-4 cells was significantly reduced by CDCA, GW4064, OCA, and FA (Fig. 4B).

### Effects of FXR-ligands on lipogenic and lipolytic gene expressions

**Fig. 5:**
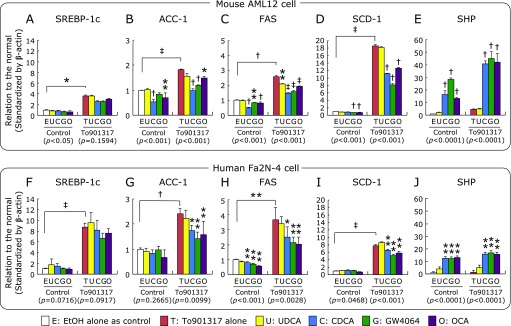
The mRNA levels of lipogenic genes; SREBP-1c (A, F), ACC-1 (B, G), FAS (C, H), SCD-1 (D, I), SHP (E, J), in AML12 and Fa2N-4 cells treated with synthetic LXRα- and FXR-ligands. Both cell lines were exposed to the various types of natural and synthetic FXR-ligand (50 µM UDCA, 50 µM CDCA, 1 µM GW4064, 1 µM OCA) and synthetic PPARα-ligand (1 µM FA) with or without 1 µM To901317 for 4 days. The mRNA levels are expressed as relative to the control following standardization by β-actin, and are shown as the mean ± SD. *p<0.05, **p<0.01, ^†^*p*<0.001 and ^‡^*p*<0.0001 shown on the column vs the respective control condition by one-way ANOVA Dunnett’s post hoc analysis, and values in the parenthesis under graph show *p* values of one-way ANOVA test. The significant difference between the control and To901317 alone was analyzed by unpaired Student’s *t* test.

In both AML12 and Fa2N-4 cells, SREBP-1c mRNA expression was significantly induced by To901317 treatment, but bile acids and synthetic FXR-ligands had no effect on the SREBP-1c mRNA level in the absence and the presence of To901317 (Fig. 5A and F). The mRNA levels of SREBP-1c target genes (ACC-1, FAS, SCD-1) were also significantly increased by To901317 treatment, and the gene inductions were significantly inhibited by CDCA, GW4064 and OCA in the both cell lines (Fig. 5B–D and G–I). On the other hand, the SHP mRNA level was significantly induced by CDCA, GW4064, and OCA in the both cell lines irrespective of the presence of To901317 (Fig. 5E and J).

**Fig. 6:**
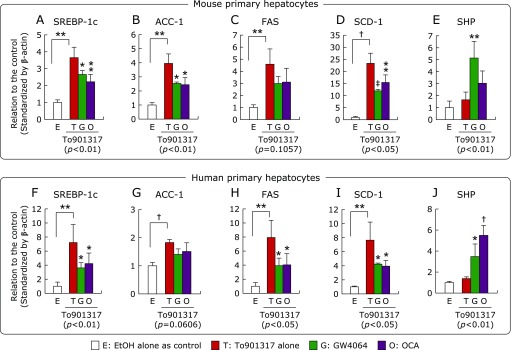
The mRNA levels of lipogenic genes; SREBP-1c (A, F), ACC-1 (B, G), FAS (C, H), SCD-1 (D, I), and SHP (E, J), in the primary hepatocytes of human and mouse treated with synthetic LXRα- and FXR-ligands. The primary hepatocytes were exposed to 1 µM To901317 with or without 1 µM GW4064 or 1 µM OCA for 24 h. The mRNA levels are expressed as relative to the control following standardization by β-actin. Data are shown as the mean ± SD from quadruplicate experiments. *p<0.05, **p<0.01, ^†^*p*<0.001 and ^‡^*p*<0.0001 shown on the column vs the To901317 alone treated condition by one-way ANOVA Dunnett’s post hoc analysis, and values in the parenthesis under the graph show *p* values of one-way ANOVA test. The significant difference between the control and To901317 alone was analyzed by unpaired Student’s *t* test.

Similar to the cell lines, the mRNA levels of SREBP-1c and its target genes were significantly increased by To901317 treatment, and the significantly increases of the lipogenic genes were significantly inhibited by the cotreatments of synthetic FXR-ligands (GW4064 and OCA) in the primary hepatocytes of human and mouse, although the significantly inhibitions were not observed in FAS in mouse and ACC-1 in human (Fig. 6A–D and F–I). The SHP mRNA level was also significantly increased by the synthetic FXR-ligands in the both primary hepatocytes (Fig. 6E and J).

**Fig. 7:**
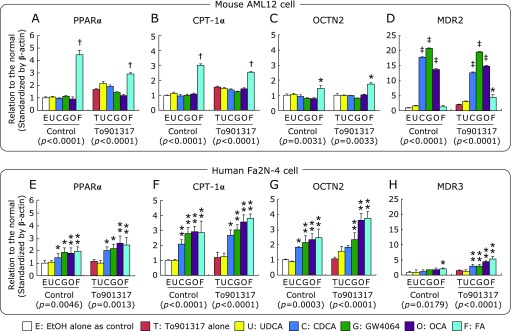
The mRNA levels of lipolytic-related genes; PPARα (A, E), CPT-1α (B, F), OCTN2 (C, G), and MDR2/3 (D, H), in AML12 and Fa2N-4 cells treated with FXR-ligands and To901317. The mRNA levels were measured by quantitative real-time PCR, standardized by β-actin, and expressed as relative to control condition. Data are shown as the mean ± SD. *p<0.05, **p<0.01, ^†^*p*<0.001, ^‡^*p*<0.0001 vs the control condition by one-way ANOVA Bonferroni’s post hoc analysis, and values in the parenthesis under graph show *p* values of one-way ANOVA test.

In AML12 cells, the mRNA levels of lipolytic genes including PPARα, carnitine palomitoyltransferase-Iα (CPT-Iα), and organic cation transporter 2 (OCTN2: SLC22A5) were significantly increased by only FA, but not the FXR-ligands, irrespective of the presence of To901317 (Fig. 7A–C). In contrast, the expression of lipolytic genes in Fa2N-4 cells were significantly increased by CDCA, GW4064, and OCA as well as FA (Fig. 7E–G).

In AML12 cells, multidrug resistance protein (MDR: ABCB4) 2 mRNA level was significantly increased by CDCA, GW4064, and OCA irrespective of the presence of To901317, and by FA in presence of To901317 (Fig. 7D). In contrast, the up-regulation of MDR3 by FXR-ligands was observed in Fa2N-4 cells, but was much less than that in AML12 cells (Fig. 7H).

### Effect of FXR-ligands on fatty acid catabolism

**Fig. 8:**
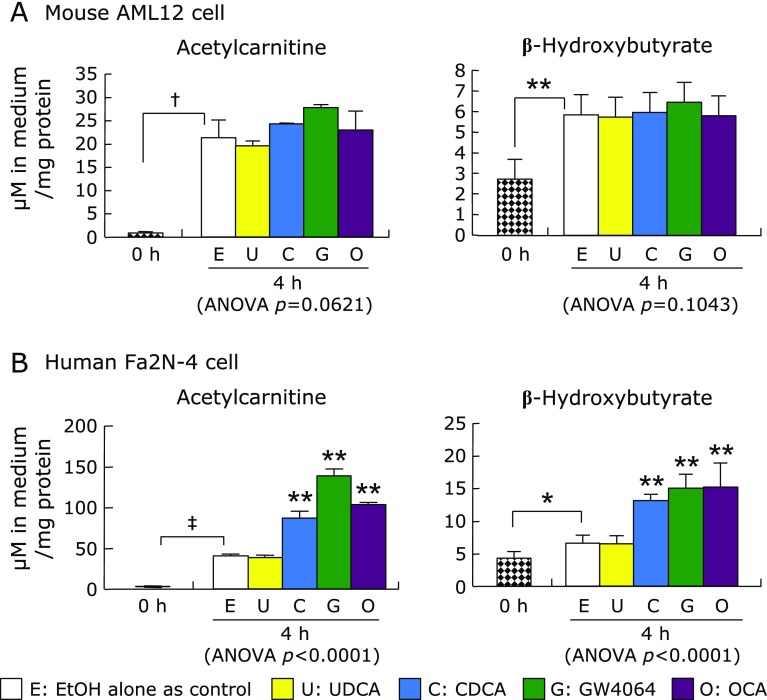
Acetylcarnitine and BHB concentrations in medium after culture cells with the natural PPARα-ligand fatty acid and the natural and synthetic FXR-ligands. AML12 and Fa2N-4 cells were incubated in the medium containing 200 µM palmitic acid, 1 mM carnitine, and FXR-ligand (UDCA, CDCA, GW4064, or OCA) or PPARα-ligand (FA) for 4 h, and then, the acetylcarnitine and BHB in the culture medium were measured by the LC-ESI-MS/MS system. Data are shown as the mean ± SD. *p<0.05, **p<0.01, ^†^*p*<0.001, ^‡^*p*<0.0001 with the bar show significant differences between the 0 h and the control condition without the nuclear receptor ligands was calculated by unpaired Student’s *t* test. **p<0.01 on the column shows the significant difference compared with the control by one-way ANOVA post hoc Bonferroni’s multiple test.

After incubation for 4 h without any treatments, the acetylcarnitine and BHB concentrations in the medium of the both AML12 and Fa2N-4 cells were significantly elevated compared with that at baseline (0 h) (Fig. 8). In AML12 cells, there were no significant difference in acetylcarnitine and BHB concentrations between the FXR-ligand treatment conditions and untreated control conditions (Fig. 8A). In contrast, both acetylcarnitine and BHB concentrations in the medium of Fa2N-4 cells were significantly increased by CDCA, GW4064, OCA, and FA (Fig. 8B).

### Effects of FXR-ligands and To901317 on cell viability

**Fig. 9:**
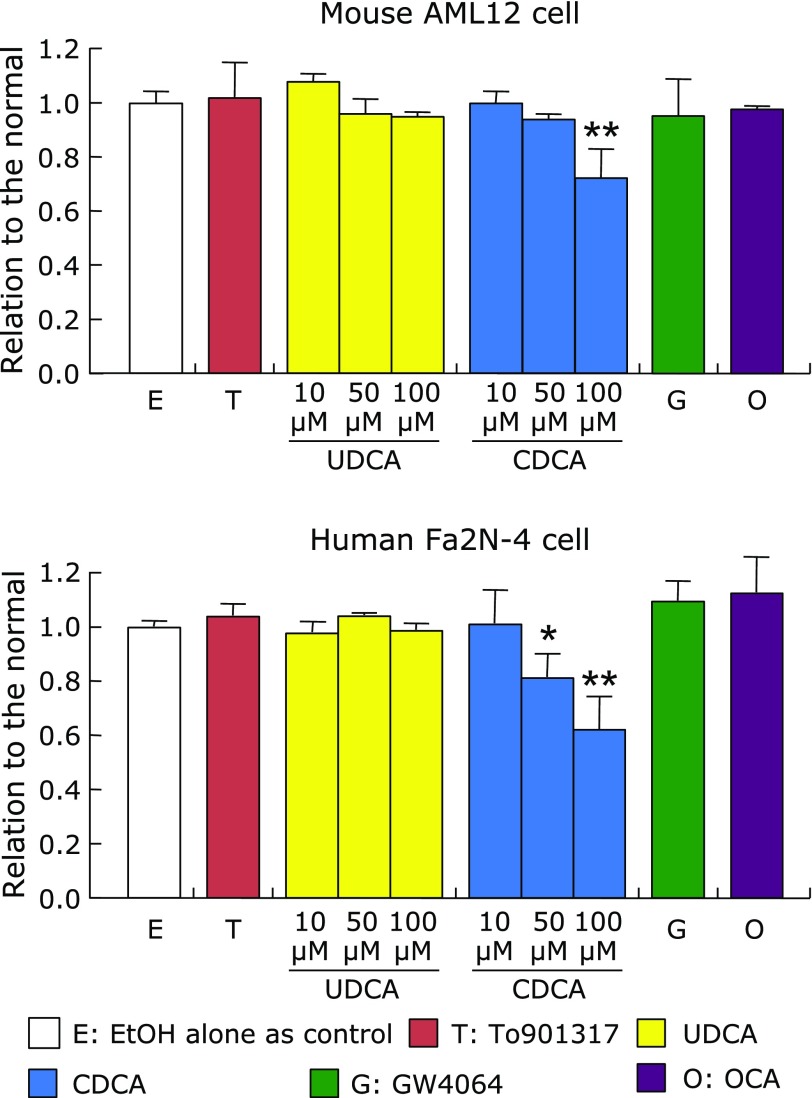
Cell viability of AML12 and Fa2N-4 cells treated with 1 µM To901317, 1 µM GW4064, 1 µM OCA, 10–100 µM UDCA or 10–100 µM CDCA for 24 h. Cell viability was measured by the MTT assay, and expressed as relative to the control condition without the nuclear receptor ligands. Data are shown as the mean ± SD. Statistical analysis was analyzed by one-way ANOVA Bonferroni’s post hoc test. *p<0.05, ^**^p<0.001, vs the control. One-way ANOVA *p* value: *p* = 0.0019 in AML12 cells, *p* = 0.0032 in Fa2N-4 cells.

Cell viability of AML12 and Fa2N-4 cells was not affected by the treatments with To901317 and FXR-ligands except for high concentrations of CDCA (Fig. 9). Fa2N-4 cell viability was reduced at 50 µM of CDCA, while that of AML12 cells was reduced at 100 µM.

## Discussion

Fatty liver is a metabolic disease that is the early stage for NAFLD and NASH with a higher risk for progression to liver cirrhosis and cancer. Therefore, early treatment of fatty liver is recommended, if early interventions including exercise and diet control that are firstly recommended are not effective. However, effective therapeutic agent for fatty liver remains to be still established. FXR ligands have been reported to improve hepatic steatosis as well as cholestasis, hepatic inflammation and fibrosis, and insulin resistance through inhibition of lipogenesis and gluconeogenesis in rodent models.^(6,20,21,23)^ The present study demonstrated that FXR activation by the natural ligand, CDCA, and by synthetic ligands, OCA and GW4064, significantly attenuated TG accumulation in a human fatty liver model through stimulation of the FXR-PPARα pathway. Furthermore, in addition to the FXR-SHP-SREBP-1c cascade, these FXR-ligand treatments significantly inhibited SREBP-1c-related lipogenesis, but did not activate PPARα-related catabolism in the mouse fatty liver model. Therefore, the FXR-ligands could be an effective therapeutic agent for fatty liver by affecting both lipid synthesis and degradation, in addition to the clinical benefits for the patients with PBC, NASH, and diabetes mellitus.^(14–17)^

An evaluation using human, but not animal cells, were needed to clarify the human-specific dual mechanisms in lipid metabolism through FXR-activation. The present study could establish the steatosis model in non-malignant cell lines derived from human (Fa2N-4) and mouse (AML12) livers by incubation with both natural and synthetic LXRα-ligands along with the induction of lipogenesis-related genes. In particular, the synthetic LXRα-ligand To901317 had more effective on the lipogenesis in a dose-dependent manner. In the To901317-induced lipogenesis phase of the steatosis model in both the human and mouse cell lines, the TG accumulation was significantly suppressed by the cotreatments of FXR-ligands in accompany with the FXR-LXRα-SREBP-1c cascade.

Present study also confirmed that there were similar gene responses to LXRα and FXR activations in the lipogenesis phase between the non-tumor hepatic cell lines and the primary hepatocytes in both mouse and human. The lipogenesis-related genes were significantly increased by To901317, and the significant increases of the genes were significantly inhibited by the cotreatments of synthetic FXR-ligands in the primary hepatocytes of both human and mouse. In addition, the SHP mRNA expression was significantly increased by the synthetic FXR-ligands in the primary hepatocytes. So, we had also attempted to establish the fatty liver model using the primary hepatocytes. In the studies using the cell lines, the lipogenesis and lipolysis phases needed the long period culture for 4 and additional 3 days, respectively. Because the primary cultured hepatocytes were hardly cultured for such long periods, it is likely to be difficult to generate the steatosis model by the LXRα-ligands.

Generally, most studies in lipid metabolism have used experimental animals induced by feeding either high fat or calorie diets, or as transgenic models (*ob/ob*, *fa/fa*).^(40)^ Data from the animal models are very valuable, but there are some species difference in lipid metabolism between humans and rodents. Furthermore, fatty acids are usually used to induce TG accumulation in culture hepatocytes,^(29,30)^ but the fatty acids-induced fatty liver model is unsuitable for the evaluation of the molecular crosstalk between FXR and PPARα because fatty acids are natural PPARα ligand.^(26,27)^ This cell culture model of fatty liver induced by LXRα-ligands should be useful for evaluating the different mechanism of lipid metabolism for each species.

The different efficiencies of ligand-induced FXR-activation on the cellular TG level between the human and mouse cells should be based on the species difference in the lipid metabolism because of the human-specific FXR-PPARα crosstalk reported by Pineda Torra *et al.*^(28)^ in addition to the universal FXR-SHP-SREBP-1c cascade.^(6)^ In the present study, the FXR-ligands (CDCA, OCA and GW4064) significantly increased the mRNA levels of PPARα and its target gene CPT-Iα in human cells in both the absence and presence of To901317, and these findings were in agreement with the study by Pineda Torra *et al.*^(28)^ Furthermore, the treatments with FXR-ligands in the human cells, but not in the mouse cells, significantly increased the mRNA level of the plasma membrane carnitine transporter OCTN2 that is also a target gene of PPARα.^(41)^ Both CPT-1α and OCTN2 are associated with carnitine, an essential molecule for fatty acid oxidation. Indeed, FXR-ligand treatment in the human cells significantly increased the extracellular excretion levels of acetylcarnitine and BHB that are metabolites of β-oxidation.^(35)^ Excess acetyl-CoA is metabolized into ketone bodies and/or acetylcarnitine when β-oxidation is up-regulated. Although OCA treatment has been previously reported to increase β-oxidation accompanied with the enhanced mRNA expressions of both PPARα and PPARγ coavtivator-1α in the skeletal muscle of Zucker rats, it is likely an indirect response through reduction of circulating free fatty acid levels because FXR is not expressed in skeletal muscle.^(20)^ The present study was the first to show that FXR-ligand treatment could directly enhance the β-oxidation reaction in human hepatocytes.

In addition, the present results showed that treatment with FXR-ligands could increase the mRNA expression of canalicular phospholipid flippase, MDR3 in human and MDR2 in mouse, to promote excretion of phospholipid into the bile, because the PPARα target genes, MDR3/2,^(42)^ are also the target genes of FXR.^(43,44)^ Indeed, the significantly increases of MDR3/2 mRNA levels by GW4064 were observed in the human hepatoma cell line,^(45)^ and in both human hepatocytes and rodent liver.^(46)^ However, the enhancement of phospholipid excretion into the bile is not likely to contribute significantly to improvement of fatty liver, because accumulated TG levels in AML cell was not influenced by FXR-ligand treatment in the present study.

In the present study, the natural bile acid CDCA, but not UDCA, showed significant effects on the fatty acid metabolism in the both human and mouse cells. These results agree with the previous study that CDCA has the most potent activity as an FXR-ligand among the natural bile acids. UDCA does not have any effect as an FXR-ligand, because the FXR-ligand activity is specific and limited to the primary bile acids, and to a much less extent to the secondary bile acids.^(47–49)^ In clinical therapy, UDCA has been used as a dissolving agent for gallstone, emollients for biliary pain, and for acute cholecystitis,^(50)^ and as a for cholestasis, including PBC.^(51)^ Although UDCA has low cytotoxicity due to its higher hydrophilicity, it does not play a role as an FXR-ligand.^(47–49)^ Based on the results in the present and previous studies, CDCA would be a candidate therapeutic agent for fatty liver as a natural FXR-ligand. CDCA has been used as therapy to dissolve cholesterol-rich gallstones,^(52,53)^ and the effects of CDCA treatment on lowering plasma TG levels were actually reported in patients with gallstones^(53)^ and with primary hypertriglyceridemia.^(54–56)^ However, higher doses of CDCA may cause some side effects, including liver damage and diarrhea due to hydrophobic toxicity. Indeed, the present study showed that the higher doses of CDCA, but not that of UDCA, caused cell damage in both human and mouse cultured cells. Therefore, it is likely that CDCA is unsuitable as a therapeutic agent for fatty liver.

On the other hand, the synthetic FXR ligand, OCA, did not cause serious adverse events except for pruritis in clinical phase I and II studies.^(14–18,57)^ OCA has a higher affinity than CDCA for the ligand-binding domain of FXR.^(7)^ Furthermore, OCA has been shown to have enterohepatic circulation. It is absorbed by the intestine and secreted into the bile after conjugations with either taurine in rodents or glycine in humans, and consequently, dramatically alter the composition of the bile acid pool by the reducing the levels of primary bile acids (choric acid and CDCA).^(14)^ The influence of reduced primary bile acids as endogenous FXR-ligands in the bile acid pool is unclear, but OCA administration has a benefit to reduce the strong cytotoxic CDCA content. In the present study, GW4064 also had an excellent activity as an FXR-ligand for improvement of fatty liver. However, there is concern that GW4064 has potential toxicity and uncertain bioavailability in humans,^(7,58)^ and therefore, it has not been tested clinically.

In conclusion, FXR-activation using either natural or synthetic ligands improved fatty liver in cultured human cells by both the decreased synthesis and increased degradation of fatty acids through human-specific dual mechanisms involving both the FXR-SHP-SREBP-1c cascade and the FXR-PPARα pathway. Because the natural FXR-ligand CDCA has a high potential to induce cellular damage, synthetic FXR-ligands, especially OCA, have already been evaluated in clinical trials and would be useful therapeutic agents as an early treatment for fatty liver.

## Author Contributions

Conceptualization, TM and AH; formal analysis, TM; investigation, TM and TI (3rd author); project administration, AH and TI (3rd author); resources, TI (4th author); supervision, YM; writing – original draft, TM; writing – review & editing, AH.

## Figures and Tables

**Table 1:** Conditions of quantitative real-time PCR and sequences of primers

Gene name	Accession number		Sequence	Product size	Elongation time
Human			(5'-3')	(bp)	(s)
ACC-1	NM_198834	F	CAG AGA CTA CGT CCT CAA GCA A	119	5
		R	GTA TGA CTT CTG CTC GCT GA		

CPT-1α	NM_001876	F	TGG AAC AGA GGC TGA AGT	192	8
		R	GGG TCT GGC TTG TTG ATA AT		

FAS	NM_004104	F	CAC CCA AGG CCA AGT ACC AT	119	5
		R	GGA TAC TTT CCC GTC GCA TA		

MDR3	M23234	F	ATA GCT CAC GGA TCA GGT C	183	8
		R	AGC ACC CAA TCC TGA GTA G		

OCTN2	NM_003060	F	CCA GAT GCT AAG AGT CAA AGG AAT	110	5
		R	GTT AGA AGG CTG TGC TTT TAA GGA		

PPARα	NM_001001928	F	CCA TCG GCG AGG ATA GTT C	144	6
		R	CGG GGA CCA CAG GAT AAG T		

SCD-1	NM_005063	F	CCG GGA GAA TAT CCT GGT TTC A	107	5
		R	TGA TGT GCC AGC GGT ACT CA		

SHP	NM_021969	F	AGA CAG ACC CCA GCC CTC C	170	7
		R	GCA CCT GCA GCA GGA GGC T		

SREBP-1c	NM_004176	F	GCC CAG GTG ACT CAG CTA TT	245	10
		R	GTC AGA GAG GCC CAC CAC TT		

β-actin	NM_001101	F	ACT GGG ACG ACA TGG AGA AA	189	8
		R	ATA GCA CAG CCT GGA TAG CA		

Mouse			(5'-3')	(bp)	(s)
ACC-1	NM_198834	F	GCT GCT GGA GGA CTT CAT	204	9
		R	AGA ATC GAC ACT GGC TGG		

CPT-1α	NM_013495	F	GGG AGA GAA TTT CAT CCA CT	198	8
		R	TGG TTT GTA TCA CTA GAG TCC A		

FAS	NM_004104	F	GGC CAC TAT ACT ACC CAA GA	112	5
		R	GAT TGT GAG CGG AAA AGT G		

PPARα	NM_011144	F	CGA CCT GAA AGA TTC GGA AAC	184	8
		R	AAG CGT CTT CTC GGC CAT A		

MDR2	NM_008830	F	ACC AGT GTC TTT TCT GAA GGT C	166	7
		R	CTG CTT CAC TGC GTC ATC		

OCTN2	NM_011396	F	TAC CAT TGA CCA GAT GCT AAG	120	5
		R	TGT TAG AAG GCT GTG CTC TTT A		

SCD-1	NM_009127	F	TG CTATCGGGGT GTTAATGA	128	6
		R	TAA CCA TGC CAC AAG ACA		

SHP	NM_011850	F	CAA GGA GTA TGC GTA CCT GA	232	10
		R	ATC TCT TCT TCC GCC CTA TC		

SREBP-1c	NM_011480	F	CCA TTG ACA AGG CCA TGC	162	7
		R	GGT CAT GTT GGA AAC CAC GC		

β-actin	X03672	F	CCT GTA TGC CTC TGG TCG TA	260	11
		R	AGA CTT CGA GCA GGA GAT GG		

ACC-1, acetyl-CoA carboxylase-1; CPT-Iα, carnitine palomitoyltransferase-Iα; FAS, fatty acid synthase; PPARα, peroxisome proliferator-activated receptor-α; MDR, multidrug resistance protein; OCTN2, organic cation transporter 2; SCD-1, steatoryl-CoA desaturase-1; SHP, small heterodimer partner; SREBP-1c, sterol regulatory element binding protein-1c; F, forward primer; R, reverse primer; bp, base pair.

## Abbreviations

ACC-1: acetyl-CoA carboxylase-1
4β-HC: 4β-hydroxycholesterol
BHB: β-hydroxybutyrate
BSA: bovine serum albumin
CDCA: chenodeoxychoric acid
CPT-Iα: carnitine palomitoyltransferase-Iα
DMEM: Dulbecco’s modified Eagle’s medium
DMEM/F12: Ham’s F12 medium
ESI: electrospray ionization
FA: fenofibric acid
FAS: fatty acid synthase
FBS: fetal bovine serum
FXR: farnesoid X receptor
25-HC: 25-hydroxycholesterol
ITS: insulin-transferrin-selenium-X
LXRα: liver X receptor-α
MDR: multidrug resistance protein
NAFLD: nonalcoholic fatty liver disease
NASH: non-alcoholic steatohepatitis
OCA: obeticholic acid
OCTN2: organic cation transporter 2
PBC: primary biliary cholangitis
PPARα: peroxisome proliferator-activated receptor-α
22R-HC: 22R-hydroxycholesterol
SCD-1: steatoryl-CoA desaturase-1
24S,25-EC: 24S,25-epoxycholesterol
SHP: small heterodimer partner
SREBP-1c: sterol regulatory element binding protein-1c
TG: triglyceride
UDCA: ursodeoxycholic acid
